# Characterization and evaluation of *Bacillus subtilis* GYUN-2311 as a biocontrol agent against *Colletotrichum* spp. on apple and hot pepper in Korea

**DOI:** 10.3389/fmicb.2023.1322641

**Published:** 2024-01-08

**Authors:** Yunjeong Heo, Younmi Lee, Kotnala Balaraju, Yongho Jeon

**Affiliations:** ^1^Department of Plant Medicals, Andong National University, Andong, Republic of Korea; ^2^Agricultural Science and Technology Research Institute, Andong National University, Andong, Republic of Korea

**Keywords:** *Colletotrichum*, *Bacillus subtilis* GYUN-2311, biological control agent, antimicrobial activity, secondary metabolites

## Abstract

Crop plants are vulnerable to a variety of diseases, including anthracnose, caused by various species of *Colletotrichum* fungi that damages major crops, including apples and hot peppers. The use of chemical fungicides for pathogen control may lead to environmental pollution and disease resistance. Therefore, we conducted this research to develop a *Bacillus subtilis*-based biological control agent (BCA). *B. subtilis* GYUN-2311 (GYUN-2311), isolated from the rhizosphere soil of an apple orchard, exhibited antagonistic activity against a total of 12 fungal pathogens, including eight *Colletotrichum* species. The volatile organic compounds (VOCs) and culture filtrate (CF) from GYUN-2311 displayed antifungal activity against all 12 pathogens, with 81% control efficiency against *Fusarium oxysporum* for VOCs and 81.4% control efficacy against *Botryosphaeria dothidea* for CF. CF also inhibited germination and appressorium formation in *Colletotrichum siamense* and *C. acutatum*. The CF from GYUN-2311 showed antifungal activity against all 12 pathogens in different media, particularly in LB medium. It also exhibited plant growth-promoting (PGP) activity, lytic enzyme activity, siderophore production, and the ability to solubilize insoluble phosphate. In trials on apples and hot peppers, GYUN-2311 effectively controlled disease, with 75 and 70% control efficacies against *C. siamense* in wounded and unwounded apples, respectively. Similarly, the control efficacy of hot pepper against *C. acutatum* in wounded inoculation was 72%. Combined application of GYUN-2311 and chemical suppressed hot pepper anthracnose to a larger extent than other treatments, such as chemical control, pyraclostrobin, TK^®^, GYUN-2311 and cross-spraying of chemical and GYUN-2311 under field conditions. The genome analysis of GYUN-2311 identified a circular chromosome comprising 4,043 predicted protein-coding sequences (CDSs) and 4,096,969 bp. *B*. *subtilis* SRCM104005 was the strain with the highest average nucleotide identity (ANI) to GYUN-2311. AntiSMASH analysis identified secondary metabolite biosynthetic genes, such as subtilomycin, bacillaene, fengycin, bacillibactin, pulcherriminic acid, subtilosin A, and bacilysin, whereas BAGEL analysis confirmed the presence of competence (ComX). Six secondary metabolite biosynthetic genes were induced during dual culture in the presence of *C*. *siamense*. These findings demonstrate the biological control potential of GYUN-2311 against apple and hot pepper anthracnose.

## Introduction

The genus *Colletotrichum* is considered one of the top 10 fungal plant pathogens with significant scientific and commercial impact (Dean et al., 2012). These fungi are highly detrimental and cause a numerous diseases including anthracnose in a variety of agricultural plants including apple and hot pepper (Than et al., 2008; Dowling et al., 2020; Guo et al., 2022). Symptoms of apple anthracnose include small, round to irregularly shaped lesions on the fruit’s surface, which are often sunken and can vary in color from tan to dark brown (Głos et al., 2022). Lesions may have a distinctive black or purple border as the disease progresses. Cracking or breaking around the lesions may also occur on infected fruits. Furthermore, the disease can cause pre-harvest fruit drop, reducing total production in apples (Kim et al., 2016).

Hot pepper anthracnose is a fungal disease that primarily affects pepper plants of the *Capsicum* species. Anthracnose can cause severe yield and quality losses in pepper crops (Than et al., 2008). Disease symptoms of hot pepper anthracnose appear as circular to irregular-shaped sunken lesions with dark centers and lighter-colored margins develop on hot peppers (Sharma et al., 2022). These lesions may enlarge over time and may also have a concentric ring pattern. Severely infected fruits may drop prematurely, resulting in reduced yields. The humid and rainy climate in Korea, particularly during the growth season, might foster the establishment and spread of anthracnose. Fungal spores thrive in high humidity and moist vegetation (Salotti et al., 2022).

*Colletotrichum* species differ in their aggressiveness and fungicide sensitivity (Munir et al., 2016; Chechi et al., 2019). Farmers currently rely extensively on synthetic fungicides to reduce crop losses caused by fungal diseases. The widespread use of these chemical fungicides, however, has led in fungicide-resistant pathogens (Ishii et al., 2022), raising concerns about their long-term impacts on the environment and human health (Książek-Trela and Szpyrka, 2022). As an alternative, the use of biocontrol agents (BCAs) to replace traditional fungicides has been advocated (Ons et al., 2020). When traditional pesticides lose their effectiveness due to the emergence of fungicide-resistant strains, using BCAs becomes quite appealing.

However, the efficacy of BCAs in disease management is frequently restricted and subject to unpredictable environmental conditions, such as temperature, humidity, and pH; which have an impact on survival of BCAs and interactions with pathogens (Fedele et al., 2020; Riseh et al., 2022). Biological control of plant diseases involves several rhizobacteria, among which *Bacillus* species is considered one of the BCAs alternative to chemical pesticides (Etesami et al., 2023). The *Bacillus* species has a high potential to control infection through direct inhibition of pathogen growth via the synthesis of several kinds of metabolites, enzymes, and low molecular-weight compounds, and volatiles (Rehman and Leiknes, 2018). The ability of *Bacillus* to grow rapidly in the medium, produces extremely tolerant endospores, and secretes various bioactive compounds that are desirable for commercialization (Wu et al., 2015). *Bacillus subtilis* complex has been reported to remain in the soil and the rhizosphere without causing any enduring damage to other bacterial populations (Radhakrishnan et al., 2017). The antagonistic activities of *Bacillus* spp. are frequently associated with the synthesis of secondary metabolites with antibiotic characteristics, including iturin group as a novel family of antibiotics isolated from *B. subtilis* (Mhammedi et al., 1982). Therefore, *Bacillus subtilis*, a well-studied Gram-positive bacterium resistant to various plant pathogens, has garnered interest in this study due to its potential as a BCA candidate for the control of anthracnose. Its ability to generate antimicrobial chemicals, compete for resources, trigger plant defense responses, and colonize the plant surface has been intensively studied (Sarwar et al., 2018; Li et al., 2019).

In light of these potential benefits, the objectives of this study is to (i) assess the potential efficacy of the selected PGPR strain GYUN-2311 as a BCA against *Colletotrichum* spp. caused apple and hot pepper anthracnose, and (ii) analyze the complete genome sequence of GYUN-2311 and identify the divergent genomic attributes.

## Materials and methods

### Antagonistic bacterium and fungal pathogens

*Bacillus subtilis* GYUN-2311, an antagonistic bacterium, was isolated from the rhizosphere soil of an apple orchard in accordance with the methodology outlined by Park et al. (2013). The selection of this bacterium was based on its demonstrated antagonistic activity *in vitro* against a wide range of bacterial and fungal phytopathogens. For long-term storage, the isolate was preserved at −80°C in tryptic soy broth (TSB) supplemented with glycerol (20%). In order to prepare bacterial suspensions, culture was grown on tryptic soy agar (TSA) plates from −80°C to 28°C for a duration of 24 h. Single colonies were then transferred to a 50-mL falcon tube containing 30 mL TSB broth. The tube was incubated at 28°C for 24 h under shaking conditions (180 rpm). The list of all fungal pathogens tested in our study is provided in Supplementary Table 1. Three fungal pathogens, namely, *C. acutatum* KACC42403, *C. coccodes* KACC48737 and *F. oxysporum* f. sp. *lycopersici* KACC40043 were obtained from Korean Agricultural Culture Collection (KACC), Agricultural Microbiology Division, RDA, Korea. The other nine were isolated in our laboratory from diseased apple fruits or ginseng roots. Fungal isolates were cultured for 7 days at 25°C on potato dextrose agar (PDA, Difco, USA) plates.

### Molecular identification of GYUN-2311

The 16S rRNA gene of the isolate GYUN-2311 was analyzed for molecular identification. The genomic DNA (gDNA) of GYUN-2311 was extracted according to the manufacturer’s instructions using a kit (BioFact Genomic DNA Extraction Kit, Biofact Co., Seoul, South Korea). The 16S rRNA gene was amplified using a *Taq* DNA polymerase in a polymerase chain reaction (PCR), with primers 27F (5′-AGA GTT TGA TCM TGG CTC AG-3′) and 1492R (5′-GGC TAC CTT GTT ACG ACT T-3′). The thermal cycling conditions were as follows: denature at 94°C for 5 min, then 35 cycles at 94°C for 30 s, anneal at 55°C for 30 s, and extend at 72°C for 1 min. The reaction mixture was held at 72°C for 10 min and then cooled to 4°C at the end of the cycle. The PCR product was purified following the manufacturer’s instructions using a PCR gel purification kit (BIOFACT Co., Seoul, South Korea). The purified PCR product was sequenced using the same primers using an automated sequencer (Genetic Analyzer 3130; Applied Biosystems, Carlsbad, CA, United States). The sequence was compared with the reference bacterial species in a genomic database using the Basic Local Alignment Search Tool (BLAST) from the National Center for Biotechnology Information (NCBI). The neighbor-joining method and the MEGA-X software (Biodesign Institute, Tempe, AZ, United States) were utilized to perform sequence alignment and phylogenetic tree construction, respectively.

### *In vitro* antagonistic activity of GYUN-2311

The *in vitro* antifungal activity of GYUN-2311 bacterial cell suspensions against a variety of fungal phytopathogens was determined (Supplementary Table 1) using a dual culture plate assay. The fungal pathogens were initially cultured for 2 weeks at 25°C on potato dextrose agar (PDA) plates, and mycelial plugs were collected for utilization in the antagonism experiments. Following that, fungal pathogen mycelial plugs (6 mm in diameter) were placed in the center of PDA medium supplemented with peptone (PDK). Sterile paper discs (6 mm in diameter) impregnated with 10 μl of GYUN-2311 bacterial cell suspensions (10^8^ CFU/mL) were placed 30 mm distant from the center of the plate on the top, bottom, right, and left sides. Sterile paper discs impregnated with TSB were served as non-treated controls. After incubation at 25°C, the growth inhibition distance between the bacterial suspension and the pathogen was measured in comparison to the untreated control. *Ds* and *Bd* 3 days after incubation; *Cfr*, *Cae*, *Cs*, *Cg*, *Fo*, and *Fs* 7 days after incubation; *Cn*, *Cfi*, and *Cc* 11 days after incubation; and *Ca* 13 days after incubation. The percentage inhibition rate was estimated from this using the following formula:

Inhibition of mycelial growth rate (%) = (1 – Mycelial growth of treatment/mycelial growth of control × 100) (Montealegre et al., 2003).

### *In vitro* evaluation of volatile organic compounds and cell-free culture filtrate from GYUN-2311 on inhibition of fungal pathogens

A sandwiched-plate assay described by Li and Kang (2022) was used to test the antifungal activity of volatile organic compounds (VOCs) produced by GYUN-2311. Fungal mycelial plugs (5 mm in diameter) taken from the 2-week-old culture were placed onto PDA plates. The antagonistic bacterium GYUN-2311 was cultured on a TSA plate for 2 days. Plates inoculated with bacteria and plates inoculated with fungal mycelial plugs were assembled, confronting each other. An additional set of fungal pathogen plates was sandwiched with TSA plates without bacterium streaks was served as a control group. All plates were sealed with a double layer of parafilm. The diameter of colonies was assessed at different incubation times: *Ds* and *Bd* after 3 days; *Cfr*, *Cae*, *Cs*, *Cg*, *Fo*, and *Fs* after 7 days; *Cn*, *Cfi*, and *Cc* after 11 days; and *Ca* 13 days following incubation at 25°C. The experiment was performed twice, in triplicate.

The antifungal activity of cell-free culture filtrate (CF) derived from GYUN-2311 was assessed by culturing GYUN-2311 bacterial cells for 5 days in different liquid media, including TSB, LB, and IND, at 28°C under shaking conditions (180 rpm). In order to acquire the CF, the culture broth was centrifuged for 5 min at 13,000 × *g* and 4°C. The resulting supernatant culture was filtered through a membrane filter (0.22 μm). The plates were spread with CF (100 μL), followed by placing the mycelial plugs (6 mm in diameter) of fungal pathogens (Supplementary Table 1) onto the center of PDK plates. PDK plates spread with TSB, LB, and IND were used as untreated controls. The growth of the following pathogenic mycelia was measured at different incubation times: 3 days for *Ds* and *Bd*; 7 days for *Cfr*, *Cae*, *Cs*, *Cg*, *Fo*, and *Fs*; 11 days for *Cn*, *Cfi*, and *Cc*; and 13 days for *Ca* following incubation at 25°C. The experiment was performed at least twice, and each treatment had three replicates (petri dishes).

### Inhibition of spore germination of *C. siamens* and *C. acutatum* by treatment with cell suspension and culture filtrate of GYUN-2311

Conidial germination and appressorium formation from *C. siamense* and *C. acutatum* were tested on a slide glass surface treated with GYUN-2311 bacterial suspensions and its culture filtrate (CF) following a method described by Kim et al. (1998). Briefly, conidia were harvested and subsequently subjected to two rinsing with ice-cold SDW from the fungal cultures grown on PDA plates for 2 weeks. On a glass slide, the conidial suspensions (10 μL) at the concentration of 10^5^ conidia/mL were combined with bacterial suspensions (10 μL) or cell-free CF. Conidia suspensions without treating bacterial suspensions or CF served as a control. The evaluation of conidial germination and the development of appressorium and primary hyphae from the pathogenic mycelia treated with GYUN-2311 were assessed during incubation at 25°C for different time periods (0, 6, 18, 24, and 48 h) in Petri dishes containing moist paper. The glass slides were observed under a light microscope (Olympus BX43, Olympus, Tokyo, Japan). All the experiments were performed twice in triplicate. The formula used to determine the conidial germination rate (%) and appressoria formation rate (%) was as follows:

Conidial germination or appressoria formation rate (%) = (germination of treated conidia/germination of control) × 100.

### Control of apple and hot pepper anthracnose by GYUN-2311 bacterial suspensions

An indoor experiment was conducted to determine the effect of GYUN-2311 bacterial suspension on apple and hot pepper anthracnose disease in wounded and unwounded apples (cv. Fuji) and hot pepper fruits (cv. Geochanghan). Apple fruits of similar size were surface-disinfected with 70% ethanol for 3 min, followed by 1% NaOCl for 3 min, rinsed three times with SDW, and air-dried in a laminar airflow chamber. Surface-disinfected apple fruits were wounded by piercing them 1 to 2 mm deep with a sterile needle and treated with GYUN-2311 bacterial suspension (10^7^ CFU/mL), or TK^®^ (Tanjeokill, a product of Koreabio, a commercially available biocontrol agent in Korea), or a mixture of GYUN-2311 and TK^®^ in a 1:1 ratio by spraying with an atomizer. Apples sprayed with SDW served as a negative control. In this experiment, nearly nine incisions were made on the surface of the fruit. After 24 h, the apples were inoculated with the spore suspensions (10^5^ conidia/mL) of *C. siamens* at wounding or unwounding sites. The fruits were kept in a plastic tray containing a wet towel at the bottom in order to maintain the humidity level at 25°C. Nine apples were used per treatment. The experiment was performed two times. The disease rate (%) and control efficacy were determined by measuring the disease index 7 days after inoculation. The disease index scale was established from 0 to 4, where 0 = no symptoms, 1 = 0–25% with disease lesions ≥ 2 mm; 2 = 26–50% with tissue showing disease lesions ≥ 4 mm; 3 = 51–75% with disease lesions < 8 mm; 4 = 76–100% with sunken lesions and spore production. The disease rate (%) and control value (%) are calculated using the following formulae:

Disease rate (%) = Σ (disease index × number of fruits with disease index)/(maximum disease index × total number of fruits) × 100.

Control value (%) = (1 - incidence rate of treatment group/incidence rate of control group) × 100.

To determine the effect of GYUN-2311 treatment on hot pepper anthracnose, fully matured and healthy hot pepper fruits were collected for the experiment. Hot pepper fruits were surface-disinfected in 70% ethanol for 3 min, immersed in 1% NaOCl for 1 min, and rinsed 2–3 times in SDW, then air-dried at room temperature. Nearly five wounds were made on the surface of the fruits with the help of a sterile needle; an additional set of unwounded hot pepper fruits was also incorporated in this investigation. Wounded or unwounded fruits were sprayed with GYUN-2311 antagonistic bacterial suspensions (10^7^ CFU/mL) or TK^®^, or a mixture of GYUN-2311 and TK^®^ in a 1:1 ratio using an atomizer (5 mL per fruit). The untreated control consisted of fruits that were treated with SDW. After 24 h, both wounded and unwounded hot pepper fruits were inoculated with 10 μL of spore suspensions (10^5^ conidia/mL) of the fungus *C. acutatum*. Following inoculation, the fruits were kept under moist conditions in plastic square plates (40 cm × 40 cm) containing filter paper. The control value (%) was derived from the disease index (DI) calculated 7 days after incubation at 25°C (DI = sum of disease ratings in fruits/total number of fruits assessed) in comparison with an untreated control. Twelve hot pepper fruits were used per treatment, and the experiment was performed two times.

### Production of lytic enzymes, siderophore production, and phosphate solubilization

The ability of bacteria to produce various enzymes, including protease, amylase, cellulase, and chitinase was assessed under *in vitro* conditions. To test the protease activity of bacteria *in vitro*, sterile paper discs (6 mm in diameter) impregnated with 10 μL bacterial suspensions were placed on gelatin media (i.e., gelatin 5 g, beef extract 3 g, protease peptone 5 g, agar 15 g, distilled water 1,000 mL) in Petri dishes. The plates were incubated at 28°C for 4 days. Plates were rinsed with following a 5 min staining with 1% tannic solution; protease activity was assessed by observing the presence of a clear halo zone surrounding the bacterial colonies, which signified the presence of hydrolyzed proteins by the bacteria. In order to assess amylase production, bacterial suspensions were inoculated onto nutrient agar (NA) plates supplemented with 0.5% soluble starch. Bacterial cell suspension-impregnated sterile paper discs (6 mm in diameter) were placed onto solid media and incubated at 28°C for 4 days. Iodine solution containing 0.3 g iodine and 0.6 g KI/L was subsequently poured into petri dishes. Clear halo zones surrounding the colonies indicated that starch degradation by bacteria was successful. To evaluate cellulase production, bacterial cell suspensions were inoculated into TSA media supplemented with 5 g/L carboxymethyl cellulose (CMC). Bacterial cell suspension-impregnated sterilized paper discs (6 mm in diameter) were placed on petri dishes at four sides at a distance of 30 mm from the edge of plates. The plates were incubated at 28°C for 4 days. Subsequently, for duration of 5–10 min. a 0.1% Congo red solution was introduced onto the surface of the petri dishes. Plates were subsequently destained with a 1 M NaCl solution. The presence of a clear halo region surrounding the colonies indicated a positive reaction to the cellulase produced by the bacteria. To determine chitinase activity, a modified method by Joe and Sarojini (2017) was used. Colloidal chitin was prepared by adding chitin powder to 12 M HCl, precipitating it with cool water, and filtering the resulting residue. This colloidal chitin was used as a screening substrate for bacteria that produce chitin-degrading enzymes. A medium was prepared with Na_2_HPO_4_, KH_2_PO4, NH_4_Cl, NaCl, yeast extract, agar, and 1% colloidal chitin. Bacterial cell suspension-impregnated sterile paper discs were placed on this medium. Following this, the plates were incubated at 28°C for 4 days. Hydrolytic clearing zones surrounding the colonies indicated chitin degradation by the bacteria.

To assess the capacity of bacterial strains to generate siderophores, the Chrome Azurol Sulfate (CAS) assay, which was originally developed by Schwyn and Neilands (1987), was utilized. A CAS solution was prepared by dissolving 121 mg of CAS in 100 mL of distilled water and combining it with a 20 mL solution of hexadecyl trimethyl ammonium bromide (HDTMA) containing 1 mM ferric chloride (FeCl_3_⋅6H_2_O) in 10 mM HCl. The CAS-HDTMA solution was sterilized before use. CAS agar plates were made by adding 100 mL of the CAS reagent to 900 mL of sterilized LB agar medium. Sterile paper discs (6 mm in diameter) impregnated with GYUN-2311 bacterial suspensions (10 μL) were placed onto CAS agar plates, and plates were incubated at 28°C for 4 days. Siderophore production was indicated by the appearance of an orange or yellow zone around the disc. The experiment was repeated once in triplicate. The ability of bacteria to utilize phosphate was assessed using the NBRIP agar plate method. Sterile paper discs (6 mm in diameter) impregnated with 10 μL of 24-h-old cultured bacterial suspensions were placed onto NBRIP agar plates. After 4 days of incubation at 28°C, distinct halo zones were observed around the paper discs. The presence of clear halos indicated that the bacteria were able to solubilize phosphate. The experiments were performed two times in triplicate.

### Growth of GYUN-2311 in different media

In ascertain the growth characteristics of the GYUN-2311 isolate across different liquid growth media, a single colony of GYUN-2311 was inoculated into a falcon tube containing 30 mL of TSB, LB [Luria-Bertani broth (LB), Difco, USA], and IND (glucose 5.45 g, L-glutamic acid monosodium salt hydrate 3.25 g, yeast extract 0.67 g, potassium phosphate 0.27 g, calcium chloride 0.27 g, cupric sulfate 0.003 g/L) (Bacto, USA) broth and incubated for 24 h at 28°C under shaking conditions at 180 rpm. Later, 1 mL (10^8^ CFU/mL) of culture broth was transferred to a 250-mL Erlenmeyer flask containing 100 mL of TSB, LB, and IND and incubated at 28°C. The samples were obtained at the following time intervals: 24, 48, 72, 96, and 120 h, and cell growth was measured by optical density at 600 nm (OD_600_ nm) using a spectrophotometer (Ultraspec 4000 Spectrophotometer; Pharmacia Biotech Ltd., Little Chalfont, Buckinghamshire, UK). To determine colony-forming units (CFU), the inoculum was serially (10-fold) diluted and spread onto TSA agar plates. The viable cells were counted 24 h after incubation at 28°C.

### Effect of antagonistic bacterium on growth promotion in hot pepper seedlings

To assess the growth-promoting potential of GYUN-2311, the hot pepper (cv. color king) seeds were planted in plastic trays (36 pots per tray) containing garden soil (Nongwoo Bio., Ltd., Yeoju, South Korea). After germination, 1-month-old hot pepper seedlings were soil drenched with 15 mL of 48-h-old cultured bacterial suspensions (10^8^ CFU/mL) of GYUN-2311. The seedlings were treated with bacterial suspensions four times at 3-day intervals. The pots were kept under greenhouse conditions, maintaining a temperature of 25 ± 5°C. An observation of growth promotion was recorded 24 days after treatment. The experiment was performed two times with 10 replicates (seedlings) per treatment, and *t*-test was used to reveal the statistical difference between the treatment and control.

### Field evaluation of the application of GYUN-2311 on disease suppression of hot pepper anthracnose

A field experiment was carried out to investigate the biological control of hot pepper anthracnose using GYUN-2311 antagonistic bacteria in Korea. Initially, the seedlings were grown in plastic trays (36 pots per tray) with one seed per pot (Kwon et al., 2022). Three-week-old hot pepper (cv. color king) seedlings were transplanted in a field at Andong National University, Andong, Gyeongbuk Province, South Korea. A spacing of 80 cm between rows and 35 cm between plants was maintained in the field. The treatments were grouped as follows: control, chemical control, pyraclostrobin (negative control), foliar spray with GYUN-2311, cross-application of chemical and GYUN-2311, and mixed spraying of chemical + GYUN-2311. The commercially available TK^®^ product was used as a positive control. All treatments used bacterial cell suspensions at a concentration of 10^7^ CFU/mL. Every 10 days the field was irrigated. The first dose was applied on July 07, 2023, and five treatments were applied at 10-day intervals until August 20, 2023, using a foliar spray method (the detailed treatment plan is shown in Supplementary Table 2). The anthracnose disease rate (%) was calculated using disease-infected hot pepper fruits harvested 1 week following the final treatment in the field. The disease rate (%) was calculated using the following formula: Disease rate (%) = Number of diseased fruits/total number of fruits × 100 (Kim et al., 2023).

### Whole-genome sequence analysis of the strain GYUN-2311

From GYUN-2311 bacterial suspensions cultured for 24 h, bacterial genomic DNA was extracted using the DNeasy Ultra Clean Microbial kit (Qiagen, Hilden, Germany) in according to the manufacturer’s instructions. The DNA contamination was assessed through the utilization of an ABI 3730 DNA sequencing machine (Applied Biosystems, Foster City, CA, United States) to sequence the 16S rRNA gene. Following confirmation of the integrity of the gDNA via agarose gel electrophoresis, sequencing libraries were generated using PacBio DNA Template Prep Kit 1.0 and BluePippin Size-Selection System, as per the manufacturer’s instructions. The 20 kb template preparation process utilized aforementioned equipment. In summary, g-tubes (Covaris, Woburn MA, United States) were utilized to shear 10 μg of the gDNA to 20 kb. Following this, the fragments underwent purification, end-repair, and ligation using blunt-end SMARTbell adapters. The libraries underwent quantification and qualification using a Qubit 2.0 fluorometer (Invitrogen, Carlsbad, CA, USA) and a DNA 12000 chip (Agilent Technologies, Waldbronn, Germany), respectively. Following that, the libraries were sequenced using PacBio P6C4 chemistry in the PacBio RSII 8-well SMART Cell v3.

The genome of the GYUN-2311 strain was generated *de novo* utilizing the HGAP2 protocol and PacBio SMRT Analysis Pipeline 8.0 with default configurations.^1^ The PacBio sequencing data were converted to Circlator 1.4.0 which was employed to prototype the resultant contigs (Pacific Biosciences, Menlo Park, CA, Inc., United States). Assembly error correction was conducted using Pilon version 1.21 (Walker et al., 2014). The complete genome assembly was assessed using the Benchmarking Universal Single-Copy Orthologs (BUSCO, v5.1.3) suite (Simão et al., 2015). Chromosome, plasmid, and contig annotation were performed using rapid prokaryotic genome annotation (Prokka, v1.13) (Seemann, 2014). Annotation of whole-genome assemblies for functional genes was performed using the EzBioCloud genome database (ChunLab Inc., Seoul, Korea).^2^ Through the utilization of orthologous group information, the coding sequences were categorized into groups (EggNOG 4.5).^3^ The genomes of Kutzneria were examined to identify potential gene clusters responsible for secondary metabolite production. This analysis was conducted with antiSMASH (version 7.0.0) (Weber et al., 2015). In addition, the BAGEL 4 online web server and BAGEL4 were used to detect potential bacteriocins in all the investigated genomes (van Heel et al., 2018).

### Gene expressions of secondary metabolites from GYUN-2311 using qPCR

The expression of secondary metabolite-related genes associated with the antimicrobial activity of GYUN-2311 was analyzed and compared using qPCR during monoculture and dual culture with *C. siamense*. The fungus was inoculated on PDA plates and incubated at 25°C for 2 weeks. The bacterial strain GYUN-2311 was cultured on TSA plates for 2 days at 28°C. The fungal mycelial plugs (6 mm in diameter) were cut using a sterile cork borer. Bacteria and fungi were cultured on both sides of PDK plates with a separation of 3.5 cm, and the plates were incubated at 25°C. The control group consisted of bacteria cultured on PDK plates at 25°C. After 5 days, cells adjacent to *C. siamense* (dual culture) and cells grown in the presence of GYUN-2311 alone (monoculture) were harvested. Total RNA was isolated from each of the collected cells using the RNeasy Mini Kit with on-column DNase I treatment (Qiagen Inc., Hilden, Germany) in accordance with the manufacturer’s instructions. In order to quantify the transcription levels of seven genes (bacillaene, bacillibactin, fengycin, subtilomycin, subtilosin A, and surfactin) in GYUN-2311 cells subjected to dual culture, quantitative reverse transcription-PCR (qRT-PCR) was performed. This analysis was conducted using CFX-96 Real-Time PCR System and a SYBR Premix Ex Taq kit from TaKaRa. The reaction mixture consisted of 1 μL of cDNA template, 5 μL of SYBR premix ExTaq™, 1 μL of each forward and reverse primer (10 mM), and 2 μL of RNase-free water in a final volume of 10 μL. The transcription levels were compared between dual culture and monoculture conditions. The sequences of seven distinct genes associated with secondary metabolites were determined based on genome sequencing data. Then, each primer was designed using the NCBI primer-BLAST algorithm, and the real-time PCR was performed, while the 16S rRNA gene was used as a control. Supplementary Table 3 contains the primer information for qRT-PCR. For each gene, relative transcript levels were estimated using the 2-Ct technique with three independent replicates.

### Statistical analysis

Analysis of variance (ANOVA) was performed on the data using version 3 of SAS JMP software (SAS Institute, Cary, NC, United States; SAS, 1995). Using the least significant difference (LSD) at a significance level of 0.05, differences between treatment means were deemed significant. All experiments were conducted at least twice, and for each experiment, the data was analyzed separately. The results of one representative experiment are provided.

## Results

### Isolation, characterization and identification of antagonistic *Bacillus subtilis* GYUN-2311

**FIGURE 1:**
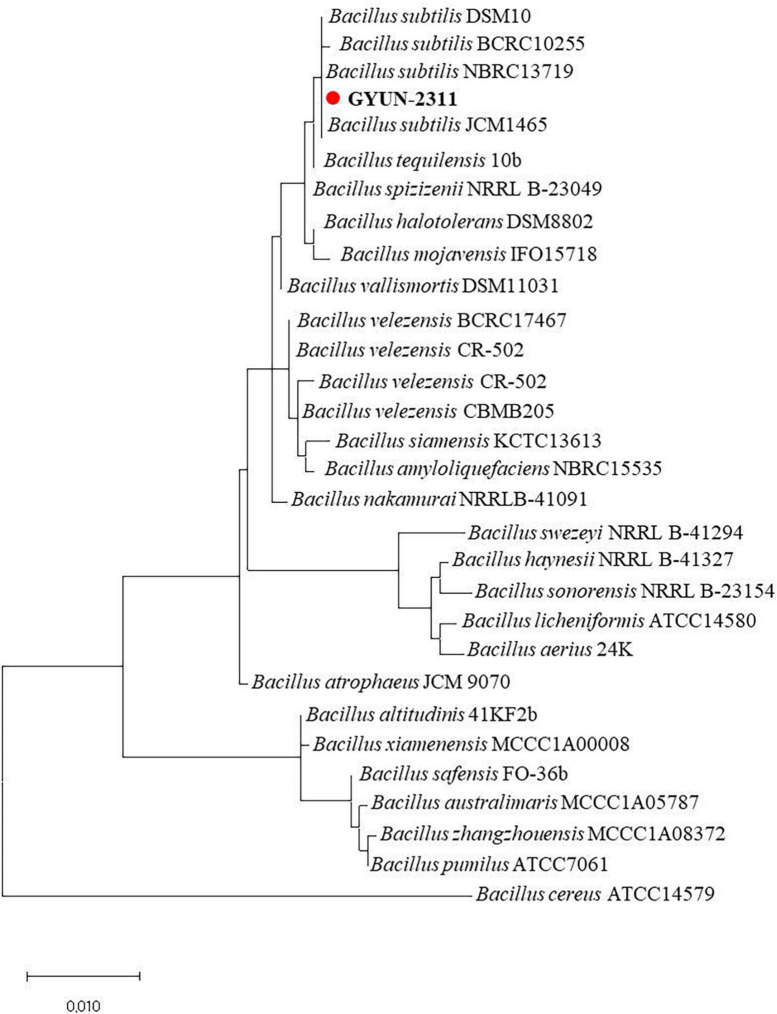
Phylogenetic dendrogram constructed from comparative analysis of 16S rRNA gene sequences showing the relationships between *B. subtilis* strain GYUN-2311 and related *Bacillus* species. Bootstrap values (expressed as percentages of 1000 replications) > 50% are shown at branch points and the species names are followed by the GenBank accession numbers. Neighbor-joining phylogenetic tree generated by the MEGA 4.0 program. The scale bar indicates 0.02 substitutions per nucleotide position.

GYUN-2311, an apple rhizosphere soil bacteria, was initially characterized using 16S rDNA gene sequencing, and the resulting sequence was submitted to GenBank under the accession number OP782591.1. BLAST analysis revealed that the GYUN-2311 isolate belonged to the *Bacillus subtilis* species, which showed high coverage (100%) and identity (99%) scores. It was 99.79% identical to *B*. *subtilis* JCM1465 (MH145363.1). The phylogenetic tree exhibited clustering of the GYUN-2311 isolate with other *Bacillus* spp., suggesting a close relationship with *B. subtilis* (Figure 1). As a result, molecular characteristics confirmed the identification of this species as *B. subtilis*. A phylogenetic tree revealed that the isolate shared near relatives with *B. subtilis* comprising other *Bacillus* species.

### *In vitro* antagonistic effect of GYUN-2311 suspensions

**FIGURE 2:**
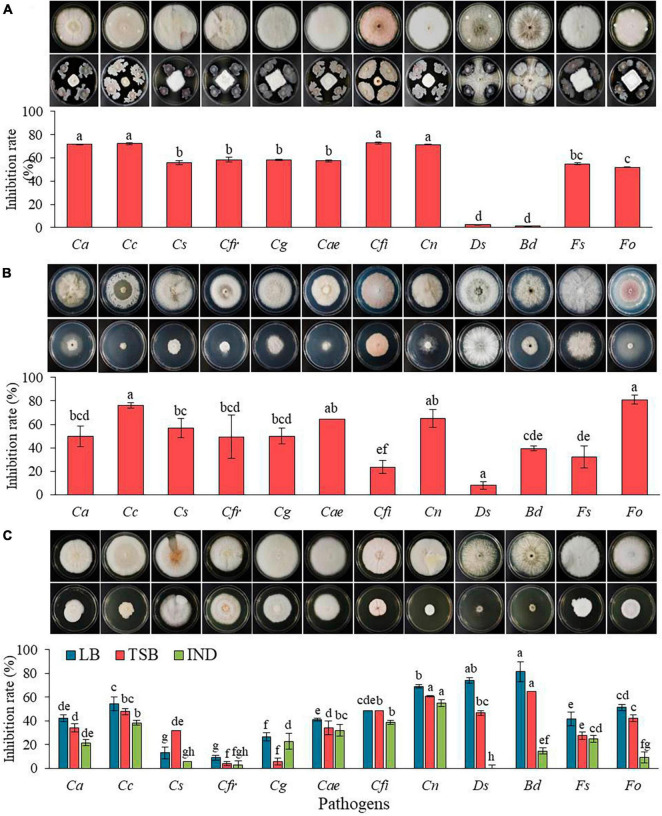
**(A)** *In vitro* antagonistic effect of GYUN-2311 against the growth of 12 fungal pathogenic isolates using a dual culture plate assay. **(B)** The inhibitory effect of volatile organic compounds (VOCs) produced by GYUN-2311 against the growth of 12 fungal pathogenic isolates was tested using the sandwiched plate assay. One set of plates streaked with the GYUN-2311 on TSA were overlapped with pathogen-inoculated plates. The colony diameter was measured at different days, such as *Ds* and *Bd* 3 days; *Cfr*, *Cae*, *Cs*, *Cg*, *Fo*, and *Fs* 7 days; *Cn*, *Cfi*, and *Cc* 11 days; and *Ca* 13 days after incubation at 25°C. **(C)** The inhibitory effect of culture filtrate of GYUN-2311 derived from three various media (LB, TSB, and IND) against the growth of 12 fungal pathogens. Plates treated with LB, TSB, and IND alone were used as a non-treated control group. The diameter of mycelial growth of fungal pathogens on PDK plates was recorded at various days (said as above) after incubation at 25°C. Each treatment consisted of three replicates (petri dishes) and the experiment was performed at least twice. Bars with the same letters do not differ from each other according to the least significant difference (LSD) at *P* < 0.05. The abbreviations representing the fungal pathogens on the X-axis are as follows: *Ca*: *Colletotrichum acutatum, Cc: Colletotrichum coccodes, CS*: *Colletotrichum siamense, Cfr*: *Colletotrichum fructicola, Cg*: *Colletotrichum gloeosporioides, Cae: Colletotrichum aenigma, Cfi: Colletotrichum fioriniae, Cn: Colletotrichum nymphaea, Ds: Diplodia seriata, Bd: Botryosphaeria dothidea, Fs: Fusarium solani*, and *Fo: Fusarium oxysporum* f. sp. *lycopersici.*

*In vitro* antagonistic activity of The GYUN-2311 bacterium against 12 fungal pathogenic isolates was assessed (Figure 2A). GYUN-2311 bacterial suspension treatment resulted in more than 50% inhibition rate against 10 pathogens. Particularly, its inhibition rate against *C. acutatum*, *C. coccodes*, *C. fioriniae*, and *C. nymphaea* was over 70%. The cell suspension treatment has been displayed minimal control effects on *Diplodia seriata* and *Botryosphaeria dothidea*. Consequently, the dual culture plate assay demonstrated a statistically significant inhibition (*p* < 0.05) of the mycelial growth of 10 fungal pathogens after treating PDK plates with bacterial suspensions of GYUN-2311. In contrast, the control group, which did not receive bacterial suspensions, exhibited no mycelial inhibition.

### *In vitro* impact of bacterial VOCs emitted by GYUN-2311 on the growth of pathogenic fungi

We investigated the inhibitory effect of volatile organic compounds (VOCs) released by GYUN-2311 bacterial cells on the mycelial growth of fungal pathogens was investigated. When the 12 fungal pathogens were exposed to VOCs emitted by GYUN-2311 bacterial colonies, fungal mycelial growth was significantly inhibited (Figure 2B). The VOCs of GYUN-2311 exhibited antifungal efficacy against all 12 fungal pathogens, with the highest inhibition rate of 81% observed against *Fo* and the lowest inhibition rate of 7.8% observed against *Ds*. The particular VOCs responsible for this behavior are yet to be identified.

### *In vitro* antifungal activity of CF of GYUN-2311

The antifungal activity of culture filtrates (CF) from GYUN-2311 against the mycelial growth of fungal pathogens was evaluated *in vitro* using LB, TSB, and IND media (Figure 2C). The CF obtained from LB demonstrated antifungal activity against all 12 fungal pathogens, with *Botryosphaeria dothidea* exhibiting the highest inhibition rate (81.4%) and *C. fructicola* demonstrating the lowest inhibition rate (8.8%). While the CF from TSB displayed antifungal activity against all 12 pathogens, but not higher than the CF from LB, with *B. dothidea* exhibiting the highest inhibition rate (64.7%) and *C. fructicola* demonstrating the lowest inhibition rate (4%). Whereas the CF from IND displayed the least antifungal activity among the CFs from three media. Except for *C. gloeosporioides*, the antifungal activity of IND filtrate against 11 fungal pathogens was lower than that of TSB. The IND filtrate had an inhibition rate of 22.7% for *C. gloeosporioides*, whereas the TSB filtrate had only an inhibition rate of 5.9%. No antifungal activity against *D. seriata* was observed. This result indicates that the CFs derived from GYUN-2311 may contain a diverse array of secondary metabolites that impede the growth of fungal pathogens when cultured in different media.

### Effect of administering culture filtrate or GYUN-2311 cell suspensions on the germination of conidia and formation of appressoria in *C. siamense* and *C. acutatum in vitro*

**FIGURE 3:**
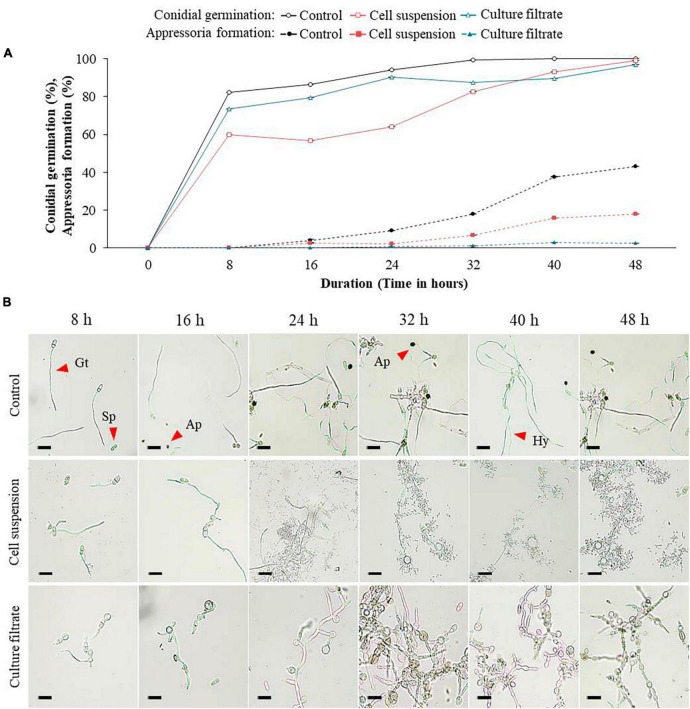
Effect of bacterial cell suspensions and culture filtrate (CF) of *B. subtilis* strain GYUN-2311 on conidia germination rate (%) and appressoria formation (%) of *Colletotrichum siamense*, and microscopic observation: **(A)** Conidial germination rate (%) and appressoria formation (%) was suppressed by bacterial cell suspensions and CF, while the germination rate (%) was increased in the non-treated control. **(B)** Microscopic observations of *C. siamense* fungal spore germination after GYUN-2311 treatment during the incubation period from 8 to 48 h. The germination counting was carried out using a hemocytometer. Bar = 10 μm. The experiment was repeated at least once in triplicates producing similar results. Abbreviations were as follows: Sp, Spore; Gt, Germ tube; Ap, Appressorium; Hy, Hyphae (bar = 10 μm).

The purpose of the experiment was to determine the effect of GYUN-2311 bacterial cell suspensions and their CF on conidial germination and appressorium formation in *C*. *siamense* and *C*. *acutatum* under *in vitro* conditions. The application of GYUN-2311 bacterial cell suspensions (10^8^ CFU/mL) or their CF to conidial spores of *C*. *siamense* resulted in varying degrees of inhibition on spore germination and appressoria formation, as compared to the untreated control group (Figure 3A). After 8 h of incubation, a hemocytometer analysis revealed a greater percentage of spore germination inhibition in the GYUN-2311 cell suspension-treated group than in the CF-treated group. It appears that the cell suspensions of the bacteria inhibited the germination of *C*. *siamense* more effectively than the CF of GYUN-2311. The control group (untreated) exhibited 82.3% germination after 8 h, while the cell suspensions-treated and CF-treated groups exhibited 60%, and 73.3% germination, respectively. The results indicate that both cell suspensions and CF inhibited the germination of *C*. *siamense* spores, with CF exhibiting a lesser effect than cell suspensions. However, after 48 h of incubation, all groups, including the control, exhibited comparable levels of conidial germination (Figure 3B). In contrast, when analyzing the effect of GYUN-2311 treatment on the appressorium formation of *C*. *siamense*, the CF had a more potent inhibitory effect than the cell suspensions treatment, with only 2.5% of appressorium formation after 48 h in the CF-treated group compared to 17.9% in the cell suspensions-treated group. The water-treated control group had the maximum level of appressorium formation (43%) after 48 h.

**FIGURE 4:**
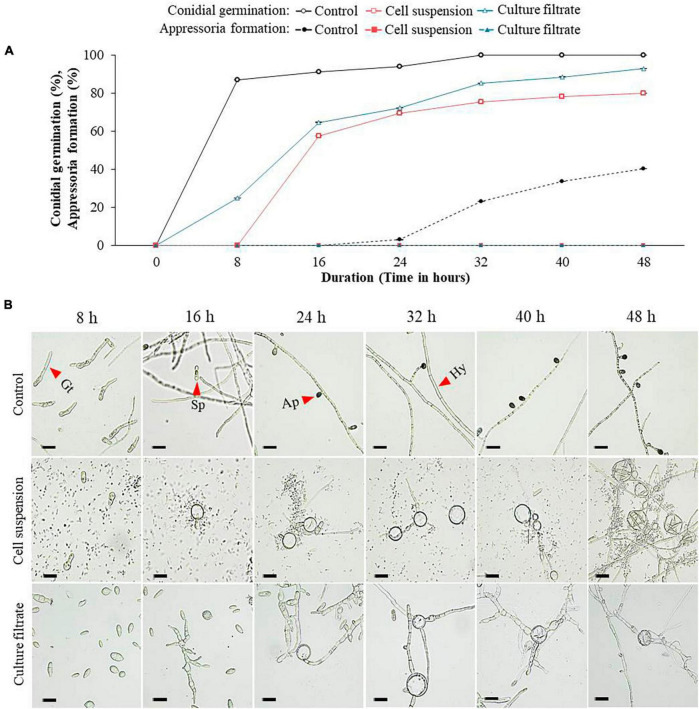
Effect of bacterial cell suspensions and culture filtrate (CF) of *B. subtilis* strain GYUN-2311 treatment on conidia germination rate (%) and appressoria formation (%) of *Colletotrichum acutatum*, and microscopic observation: **(A)** Conidial germination rate (%) and appressoria formation (%) were suppressed by bacterial cell suspensions and CF, while the germination rate (%) was increased in the non-treated control. **(B)** Microscopic observations of *C. acutatum* fungal spore germination after GYUN-2311 treatment during the incubation period from 8 to 48 h. The germination counting was carried out using a hemocytometer. Bar = 10 μm. The experiment was repeated at least once in triplicates producing similar results. Abbreviations were as follows: Sp, Spore; Gt, Germ tube; Ap, Appressorium; Hy, Hyphae.

Comparatively, when the effect of GYUN-2311 treatment on *C. acutatum* spore germination (%) and appressorium formation (%) was examined, varying degrees of inhibition was observed compared to the untreated control (Figure 4A). The results demonstrated that both bacterial cell suspensions and CF inhibited the conidial germination of *C. acutatum*, with cell suspensions having a more potent effect than CF. After 8 h of incubation, the control group had a germination rate of 87%, whereas the groups treated with cell suspensions and CF had germination rates of 0 and 25%, respectively. In both the CF and cell suspensions, the appressorium formation was 0% after 48 h (Figure 4B). In the experiment, the fungus also developed gall-like structures on its hyphal wall, suggesting that GYUN-2311 was involved in inhibiting the germination of *C. acutatum* and *C*. *siamense* conidia. The CF from GYUN-2311 exhibited a significant (*P* < 0.05) inhibitory effect on the appressorium development of *C*. *siamense* and *C. acutatum*. In contrast, living cells demonstrated a more effective inhibitory effect on the germination of *C*. *siamense* or *C. acutatum*.

### Control of apple and hot pepper anthracnose by GYUN-2311 suspensions

**FIGURE 5:**
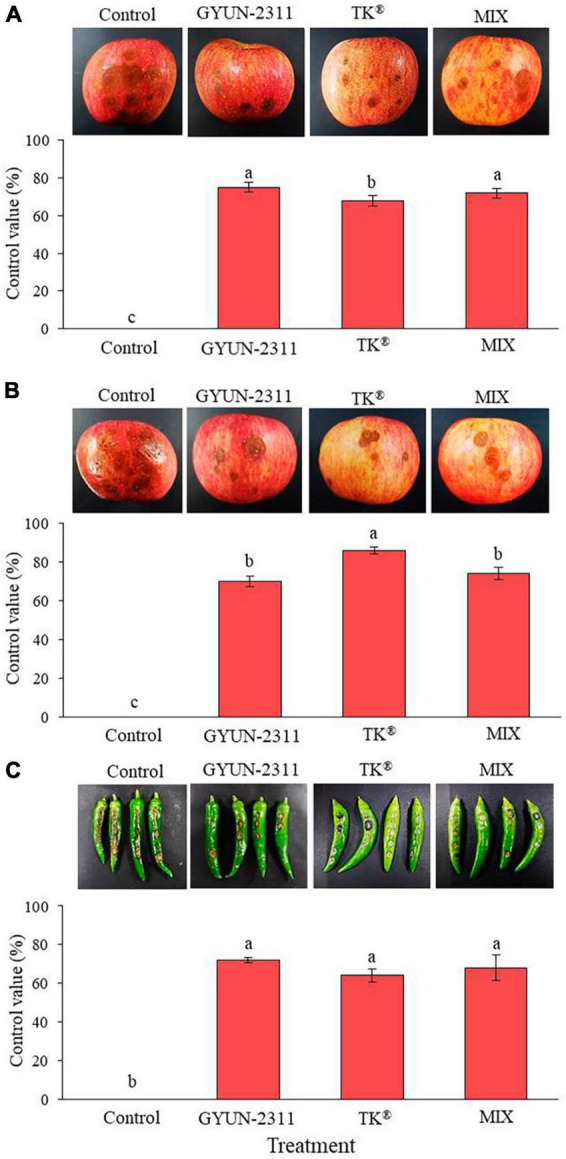
Effect of GYUN-2311 bacterial cell suspensions on control of anthracnose caused by *C. siamense* in wounded **(A)** and unwounded **(B)** apples under *in planta* conditions. Apples treated with SDW served as a control. The experiment was performed two times and each treatment consisted of 12 replicates (apples). **(C)** Effect of GYUN-2311 bacterial cell suspensions on control of anthracnose caused by *C. acutatum* in hot peppers under *in planta* conditions. Hot pepper fruits treated with SDW served as a control. The experiment was performed two times and each treatment consisted of 12 replicates. The results were compared with a non-treated control 7 days after incubation at 25°C. Bars with the same letters do not differ from each other according to the least significant difference (LSD; *p* < 0.05).

The effectiveness of GYUN-2311 in protecting apples from anthracnose caused by *C. siamense* was evaluated in both wounded and unwounded inoculations. GYUN-2311 demonstrated a control value of 75% against *C. siamense* in the wounded inoculation, whereas the commercial product TK^®^ attained a control value of 67.81%. Combining GYUN-2311 and TK^®^ increased the control value to 72% (Figure 5A). In the non-wounded inoculation, GYUN-2311 had a control value of 70% against *C. siamense*, while TK^®^ had an 86% control value, and the combination of GYUN-2311 and TK^®^ had a 74% control value. Consequently, the preventive effect of GYUN-2311 suspension was comparable for both wounded and unwounded inoculations against *C. siamense*. In the wounded inoculation, GYUN-2311 exhibited a higher control value than TK^®^ (Figure 5B). In the case of the preventive effect of GYUN-2311 (10^7^ CFU/mL) to control hot pepper anthracnose, caused by *C. acutatum*, the GYUN-2311 treatment on wounded hot peppers exhibited the highest control value of 72% against *C. acutatum*, which was greater than the control values of TK^®^ with 64% and the combined treatment of GYUN-2311 and TK^®^ with 68% (Figure 5C). This finding indicates that the bacterium GYUN-2311 can be recommended as one of the biocontrol agents to reduce the incidence of anthracnose in apples and hot peppers.

### Effect of GYUN-2311 bacteria on lytic enzyme production, siderophores, and phosphate solubilization

To investigate the enzymatic activities associated with the potent antimicrobial properties of GYUN-2311, we analyzed the activities of lytic enzymes, such as protease, cellulase, chitinase, and amylase (Supplementary Figures 1A–D). GYUN-2311 exhibited robust enzymatic activity in each of the four enzymes tested. Furthermore, the qualitative assessment of GYUN-2311 to produce siderophores was evaluated using a CAS assay. A positive qualitative assay is characterized by the transformation of the CAS agar medium surrounding the inoculation site from blue to orange. Our results indicated that GYUN-2311 exhibited a moderate level of siderophore production by the formation of distinct halo zones on the CAS agar medium (Supplementary Figure 1E). The findings of this study indicate that the bacterium produced siderophores that facilitated the binding to Fe^3+^ ions to dissolved iron facilitated competition with pathogens for iron, a vital element for survival. In addition, it exhibited a moderate capacity to solubilize insoluble phosphate, as evidenced by the presence of faint halo zones on media containing tricalcium phosphate (Supplementary Figure 1F).

### Optimization of growth media for culturing GYUN-2311, and its effect on plant growth promotion

**FIGURE 6:**
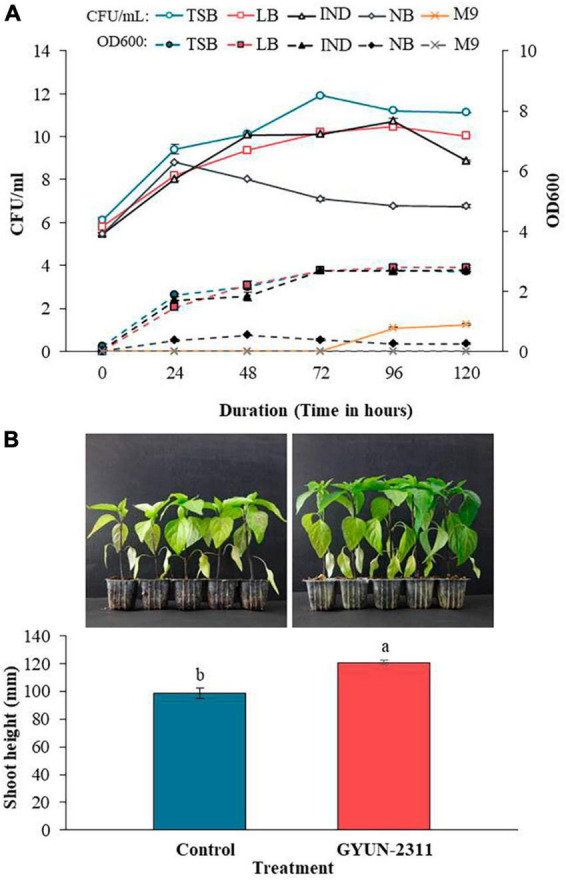
**(A)** Effect of various growth media on GYUN-2311. Optical density (OD) was read at 600 nm and colony forming units (CFU) were determined at 24 h intervals for 120 h duration of time. The experiment was repeated once. TSB, tryptic soy broth; LB, Luria-Bertani broth; IND, industrial medium; NB, nutrient broth, M9, minimal broth. **(B)** Effect of GYUN-2311 treatment on growth promotion of hot pepper. Effect of treatment with GYUN-2311 bacterial suspensions (10^8^ CFU/mL) on growth promotion of hot pepper seedlings in comparison to non-treated control (water) under greenhouse conditions. The experiment was repeated at least once with 10 replicates (seedlings) per treatment. Bars with the same letters do not differ significantly between each other according to the least significant difference (LSD; *p* < 0.05).

Bacterial cells of GYUN-2311 were found to be more abundant in TSB, with the maximum colony count at 72 h, compared to LB and IND (Figure 6A). In contrast, the colony count and OD values in NB were decreased after 24 and 48 h, respectively. Even though colonies were visible on M9 agar after 72 h, the OD value remained unchanged at 120 h. GYUN-2311 did not grow on M9 agar but thrived on TSB, LB, and IND media. TSB had a concentration of 10^9^ CFU/mL after 24 h, while IND required 48 h to reach the same concentration. The plant growth-promoting (PGP) activity of GYUN-2311 was observed in hot pepper seedlings under greenhouse conditions (Figure 6B). The GYUN-2311-treated seedlings exhibited a significant increase in height relative to the untreated control seedlings, with the difference becoming apparent as early as 6 days after the treatment. By the end of the 24-day observation period, the difference in height obtained by the treatment between the two groups had increased substantially, with the control group having a height difference of 99 mm, while the group treated with the cell suspension of GYUN-2311 had a height difference of 121 mm.

### Effect of GYUN-2311 on control of hot pepper anthracnose in field conditions

**FIGURE 7:**
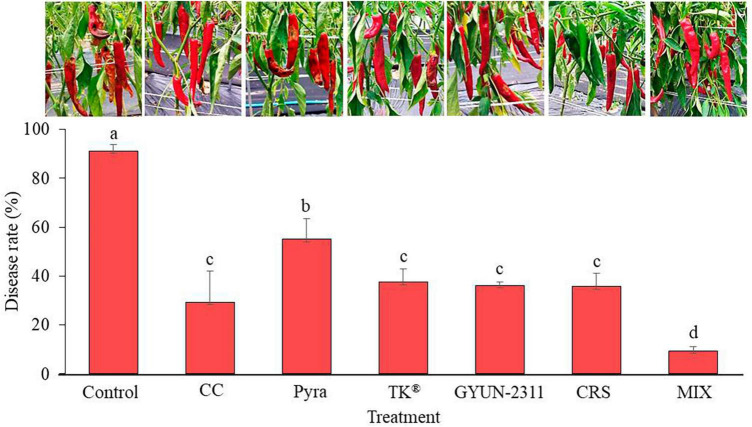
Suppression of anthracnose by treatment with GYUN-2311 under field conditions. After transplanting 3-month-old hot pepper seedlings in the field, the plants were treated with GYUN-2311, cross-spraying GYUN-2311 and chemical, mixed spraying of chemical + GYUN-2311, control (water), chemical control, pyraclostrobin (negative control) and TK^®^ five times in 50 d using a foliar spray method. Disease rate (%) was recorded from disease-infected hot pepper fruits after 7 days of the last treatment. The data for GYUN-2311 were compared with the control. For all the treatments, three plots with 20 replicates (plants) were used. CC: chemical control, Pyra: pyraclostrobin, TK^®^ : *Bacillus velezensis* AK-0, G2311: GYUN-2311, CRS: cross-spraying of chemical and GYUN-2311, MIX: Mixed spraying of chemical and GYUN-2311.

The ability of bacterium GYUN-2311 to inhibit hot pepper anthracnose, which is caused by *C. acutatum*, was evaluated in field conditions. The application of GYUN-2311 treatment resulted in a notable decrease in anthracnose (*p* < 0.05). The disease rate for those who received GYUN-2311 treatment alone was 36.19%. Whereas cross-spraying of chemical and GYUN-2311 or mixed spraying of chemical + GYUN-2311 exhibited 35.74 and 9.34% disease rate, respectively, 1-week after the last treatment (Figure 7). Chemical control, pyraclostrobin, and TK^®^ have been displayed to show 29.31, 55.03, and 37.52% disease rate, respectively. In general, the efficacy of treatments incorporating a combination of chemical spraying and GYUN-2311 in controlling disease rate (%) was superior to that of the chemical treatments alone and the untreated control. GYUN-2311 treatments reduced disease incidence more than pyraclostrobin.

### Genome analysis of GYUN-2311 and comparison with *Bacillus strains*

**FIGURE 8:**
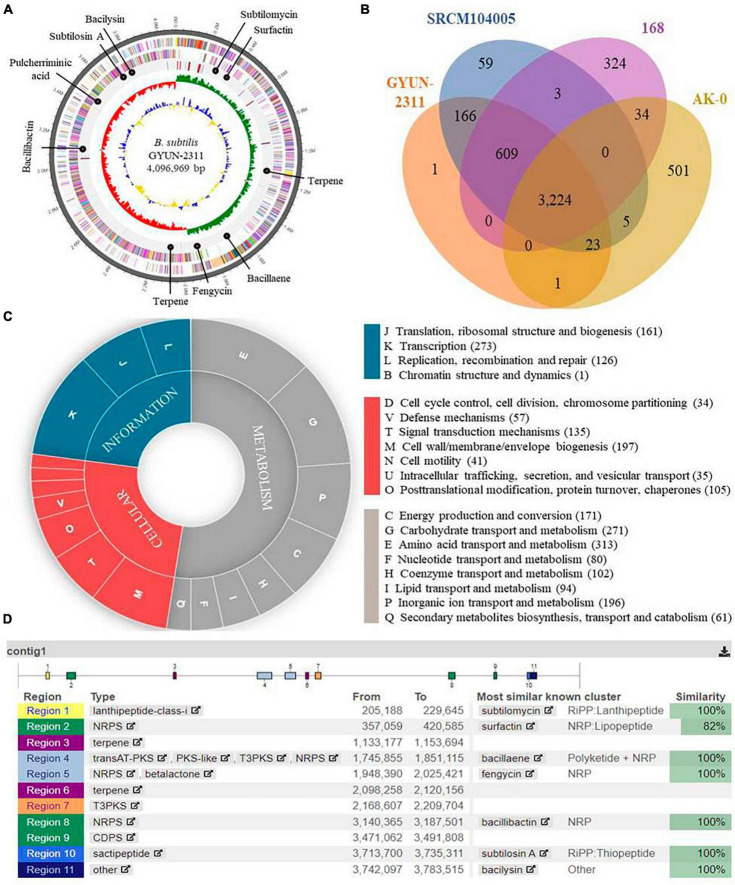
Whole-genome map of *Bacillus subtilis* GYUN-2311 and annotation. **(A)** Marked characteristics shown from the outside to the center; coding sequence (CDS) on forward strand, CDS on reverse strand, tRNA, rRNA, guanine-cytosine (GC)-content, and GC skew. CDS genome consists of a single circular chromosome that is 4.0 mb in size, and represents one contig from the outer part of the circle; the second circle represents forward, the third represents reverse strain, and the fourth circle represents tRNA and rRNA positions; **(B)** Distribution of orthologous genes in the *B. subtilis* GYUN-2311, *B. velezensis* AK-0, *B. subtilis* strain 168^T^ and *B. subtilis* strain SRCM104005 genomes. The Venn diagram shows the summary of unique SNPs from total genes of the *B. subtilis* GYUN-2311 strain. This analysis exploits all CDS of the genomes and is not restricted to the core genome; **(C)** the clusters of orthologous genes (COG) function annotation of *B. subtilis* GYUN-2311, and distribution of genes in different COG function categories; and **(D)** analysis of secondary metabolites of *B. subtilis* GYUN-2311 using the antiSMASH program.

Based on NCBI Prokaryotic Genomes Automatic Annotation Pipeline (PGAAP) analysis, the genome of GYUN-2311 was a circular chromosome with 4,096,969 bp, containing 4,043 predicted protein-coding sequences (CDSs) in 4,043 genes, 86 tRNA genes, 30 rRNAs, and an average G + C content of 43.8% (Figure 8A). The comparative Venn diagrams (Figure 8B) illustrate the shared CDSs of *B. subtilis* GYUN-2311, *B. velezensis* AK-0, *B. subtilis* SRCM104005, and *B. subtilis* 168T. This segment presents a comprehensive overview of the distinct protein-coding genes that comprise the total genes of GYUN-2311. There were 3224 common high-expression gene families shared by the four strains (GYUN-2311, AK-0, SRCM104005, and 168T), while 1, 59, 324, and 504 genes were determined to be unique for GYUN-2311, SRCM104005, 168T, and AK-0, respectively. To determine the degree of dissimilarity between *B*. *subtilis* GYUN-2311 and other *Bacillus* strains, a comparison was made between the genome of GYUN-2311 and the complete genomes of five *Bacillus* strains, namely *B*. *subtilis* SRCM104005, *B*. *subtilis* 168T, *B*. *subtilis* MBI 600, *B*. *subtilis* XF-1, and *B*. *velezensis* AK-0 (Table 1).

**TABLE 1:** Comparative genome analysis of *Bacillus subtilis* GYUN-2311 with other *Bacillus strains*.

	Taxon name	Strain name	No. of contigs	Genome size (bp)	DNA G + C content (%)	No. of CDSs	No. of rRNA genes	No. of tRNA genes
1	*Bacillus subtilis*	GYUN-2311	1	4,096,969	43.8	4,043	30	86
2	*Bacillus subtilis*	SRCM104005	1	4,136,114	43.8	4,113	30	86
3	*Bacillus subtilis*	168	1	4,215,606	43.5	4,220	30	86
4	*Bacillus subtilis*	MBI 600	1	4,076,736	43.8	4,020	30	86
5	*Bacillus subtilis*	XF-1	1	4,061,186	43.9	4,036	27	77
6	*Bacillus velezensis*	AK0	1	3,969,429	46.5	3,808	27	86

### Constraints and functionality of genome annotation

Genome annotation facilitated the functional classification of predicted protein sequences in the GYUN-2311 genome. This process involved comparing protein sequences to the COG database to identify similar amino acid sequences with similar properties. Each protein was assigned a COG number based on its functional classification. Figure 8C depicts the distribution of genes throughout the functional categories of the COG. The gene ontology pie chart depicts the cellular function (red), information (blue), and metabolism (gray) of the predicted genes derived from the assembled genome. There were 966 genes labeled “function unknown”, and 611 genes labeled “general function prediction only” among the total number of genes. However, after excluding these genes, a total of 2,453 genes were successfully assigned to COG families, which spanned across 23 functional categories. It is worth noting that the largest group of genes associated with amino acid transport and metabolism consisted of 313 genes. In addition, 61 proteins were involved in secondary metabolite biosynthesis, indicating that GYUN-2311 produced a substantial amount of secondary metabolites, particularly antibiotics. Comparative genomic analysis of GYUN-2311 revealed that the strain shares 4,022 POGs with the *B*. *subtilis* SRCM104005 strain, while it shares 3,248 POGs with the *B*. *velezensis* AK-0 strain and possesses 776 unique POGs (Figure 8B).

### Secondary metabolites of *B. subtilis* GYUN-2311 using antiSMASH

To identify highly promising genes associated with the biosynthesis of secondary metabolites in bacterial genomes, antiSMASH version 7.0.0 was employed for the rapid genome-wide identification, annotation, and analysis of gene clusters (BGCs) involved in secondary metabolite biosynthesis in GYUN-2311.^4^ It has been possible to identify eleven gene clusters that are involved in the biosynthesis of secondary metabolites. Three of these clusters are involved with the generation of non-ribosomal peptides; including bacillibactin, fengycin, and surfactin (Figure 8D). One cluster is responsible for the biosynthesis of polyketide, specifically bacillaene. Two clusters are responsible for terpene biosynthesis, while two others encode for ribosomal peptides, namely bacilysin, subtilosin A, and subtilomycin. In addition, there is one cluster responsible for pulcherriminic acid and another cluster encoding t3pks (a cluster whose identity is unknown). A fascinating discovery is that the GYUN-2311 genome contains multiple distinct gene clusters (BGCs), a number of which share 100% sequence similarity with previously identified biosynthetic gene clusters in MIBiG. These perfectly matched BGCs have been associated with the production of subtilomycin, bacillaene, fengycin, bacillibactin, pulcherriminic acid, subtilosin A, and bacilysin. In addition, a BGC involved in surfactin biosynthesis shared a similarity sequence of 82%. The BAGEL 4.0 analysis effectively identified three potential gene clusters in GYUN-2311 (Supplementary Figure 2A). These gene clusters were located within specific areas of interest (AOI) as determined by the program. AOI 1 was associated with competence (ComX), AOI 2 exhibited the greatest similarity to subtylomycin (Lanthipeptide), and AOI 3 was a match for subtilosin A (Sactipeptide). The ANI values were calculated using Ezbiocloud (Lee et al., 2016; Yoon et al., 2017). Among the *Bacillus* genomes, the closest strain to GYUN-2311 was *B*. *subtilis* SRCM104005 with ANI of 99.97%. As anticipated, *B*. *velezensis* AK-0 was the most distant strain, with ANI of 77.30%. The OrthoANI dendrogram illustrates the relationship between the six *Bacillus* strains (Supplementary Figure 2B).

### Quantification of secondary metabolites gene expression in GYUN-2311 through qPCR

**FIGURE 9:**
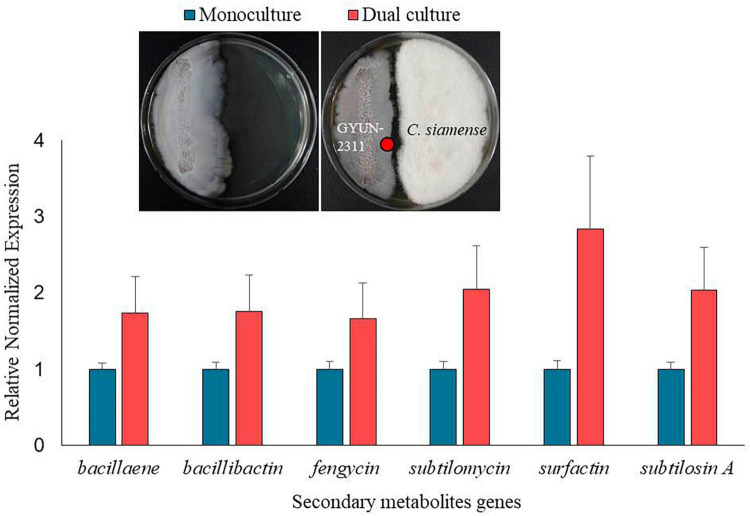
Comparison of gene expression of 6 secondary metabolites between monoculture and co-culture conditions. The red dots on the plate image represent the sampling points for RNA extraction.

The high antagonistic activity of GYUN-2311 suggests its potential to produce secondary metabolites during interactions with pathogens. GYUN-2311 genomic study revealed the gene sequences involved in secondary metabolite production. The transcription levels of six genes involved in secondary metabolite biosynthesis (bacillaene, bacillibactin, fengycin, subtilomycin, subtilosin A, and surfactin) were evaluated using quantitative reverse transcription-PCR in dual culture and monoculture conditions. These genes showed 1.67–2.83 times higher expression in dual culture with the pathogenic fungus *C. siamense* than in monoculture (Figure 9).

## Discussion

Anthracnose causes significant annual losses on farms, and the introduction of pesticide-resistant forms exacerbates the problem (Kim et al., 2021). Biological control has the potential to be an alternative method. The goal of this study is to investigate the effectiveness of a biological control agent (BCA) against anthracnose-causing fungus in apples and hot peppers, as well as growth-promoting capability of the PGPR strain. *Bacillus* species are common bacteria with the potential to produce a wide spectrum of antibiotics (Koilybayeva et al., 2023). These antibiotics can be used to control plant diseases instead of synthetic pesticides and chemical fertilizers. Therefore, in this study, we investigated the antagonistic activity of *B. subtilis* GYUN-2311 strain against 12 fungal pathogens. Among these, three were identified to be prominent pathogens causing crop losses: *C. acutatum*, responsible for hot pepper anthracnose; *C. siamense* and *C. fructicola*, responsible for apple anthracnose. Prior investigations (Lee et al., 2012; Shi et al., 2021; Park et al., 2022) have established that a number of *B. subtilis* strains possess antagonistic properties against a range of *Colletotrichum* species, including *C. acutatum* and *C. siamense*. The antagonistic activity of bacteria is mediated via synthesis of a variety of volatile or non-volatile secondary metabolites (Kai et al., 2009; Torres et al., 2015). Almeida et al. (2023) report that volatile organic compounds (VOCs) generated by various bacterial species contribute to disease suppression. Several *Bacillus* species produce VOCs with antifungal properties, protecting crops from several phytopathogenic fungi (Caulier et al., 2019; Guevara-Avendaño et al., 2019). VOCs produced by some other bacterial species, such as *Pseudomonas* spp. exhibit antifungal effects against *Rhizoctonia solani*, resulting in plant disease reduction in rice (Wang et al., 2021). Several rhizobacteria-produced volatiles have been reported to exhibit antibacterial or antifungal properties (Kai et al., 2009). These findings complement our sandwich plate findings that VOCs from the strain GYUN-2311 suppressed fungal mycelia of several pathogens. GYUN-2311 also affected the 12 pathogens evaluated in this study. GYUN-2311 inhibited *F. oxysporum* (*Fo*) by 81.02% using just VOCs; more than 60% of *Cc*, *Cn*, *Cae*, and *Cg* were inhibited. Despite the fact that the specific VOCs produced by GYUN-2311 have not yet been identified, it is evident that they possess significant antimicrobial activity.

We evaluated the activity of the GYUN-2311 CF against the mycelial growth of 12 fungal pathogens obtained from three different media (LB, TSB, and IND), with the CF from LB demonstrating the highest level of growth inhibition activity. This is owing to variations in secondary metabolites across different media. Antifungal proteins and lipopeptide antibiotics are among the antifungal components found in CFs of *Bacillus* (Torres et al., 2015). By interfering with spore activity, causing damage to pathogenic hyphae, and enhancing host resistance activities, these antifungal components resist pathogen invasion via multiple mechanisms (Yánez-Mendizábal and Falconí, 2021). The *in vitro* results demonstrated that GYUN-2311 inhibited spore germination and mycelial growth in *C*. *siamense* (*Cs*) and *C*. *acutatum* (*Ca*) to a significant (*P* < 0.05) level. In particular, CF alone effectively inhibited spore germination and mycelial growth in *Cs* and *Ca*. CF is more effective at inhibiting appressorium formation, while GYUN-2311 cell suspension is more effective at inhibiting conidial germination. The presence of diverse antifungal compounds may account for the inhibitory effect of CF (Ongena and Jacques, 2008). CF contains extracellular compounds that are secreted by growing bacterial cells (Heo et al., 2022). In contrast, bacterial cells in cell suspension may compete with fungal cells for nutrients or space, produce antifungal compounds, or activate the host plant’s defense mechanisms (Ongena and Jacques, 2008).

Our study has investigated the efficacy of *B. subtilis* as a biocontrol agent targeting *Colletotrichum* species, highly destructive pathogens affecting various crops, including hot peppers and apples. *B. subtilis* has been shown to reduce anthracnose infections caused by *C. gloeosporioides* in red peppers (Lee et al., 2020) and *C. acutatum* in apples (Kim et al., 2015). Furthermore, a number of *B. subtilis* strains have been documented to impede the development of diverse fungal pathogens at varying degrees. For instance, Ku et al. (2021) observed that *B. subtilis* C3 inhibited the germination of *F. oxysporum* spores. Guo et al. (2014) identified *B. subtilis* NCD-2 as an inhibitor of *Rhizoctonia solani* growth. *B. subtilis* M4 involved in the suppression of *Pythium ultimum* (Ongena et al., 2005), and *B. subtilis* Z-14 has been reported to suppress the growth of *Botrytis cinerea* (Chen et al., 2019). Many studies have shown that a range of antifungal compounds generated by *Bacillus* spp. are important in biocontrol activity (Cazorla et al., 2007; Hashem et al., 2019). Furthermore, the synthesis of additional enzymes, such as amylase (Dai et al., 2020), protease and cellulase by *Bacillus* spp. may contribute to the suppression of diseases caused by pathogenic fungus (Sritongon et al., 2023). Chitinases produced by *B*. *subtilis* have antifungal properties and can degrade the cell walls of several pathogenic fungi, including *R. solani* (Saber et al., 2015). It was determined that GYUN-2311 produces proteases, cellulases, chitinases, and amylase, in addition to siderophore and phosphate solubility. It is essential to note, however, that the efficacy of *B. subtilis* varies depending on the strain used, environmental conditions, and type of pathogenic fungus. Therefore, the use of *B. subtilis* as a biocontrol agent must be carefully evaluated using substantial experimental data. Furthermore, additional research is required to understand the mechanisms of action and signaling pathways associated with secondary metabolites against pathogenic fungi.

The strain GYUN-2311 has been found to promote the growth of hot pepper seedlings in greenhouse conditions, which is supported by the ability of the strain to produce siderophores. These siderophores play a vital role in the accumulation of Fe from various organic materials (Sharma and Johri, 2003) are iron-chelating compounds produced by rhizobacteria that can enhance plant growth by increasing the availability of iron in the soil (Yu et al., 2011) and suppress the pathogen growth by limiting iron availability (Singh et al., 2022). *B. subtilis* is known to produce a variety of enzymes capable of degrading plant cell walls and inhibiting the growth of plant pathogens (Hashem et al., 2019).

Combined application of GYUN-2311 and chemical suppressed hot pepper anthracnose more effectively than other treatments, such as chemical control, pyraclostrobin, TK^®^, GYUN-2311, and cross-spraying of chemical and GYUN-2311 under field conditions. On the other hand, the GYUN-2311 treatment has shown the disease rate of 36.18% which is similar to TK^®^ treatment with disease rate of 37.52%. In support of our findings, Suprapta (2022) provided evidence that the application of *Paenibacillus polymyxa* C1 successfully reduced the occurrence of anthracnose incidence in chili peppers under greenhouse conditions by 30.38–97.34% subsequent to treatment with different formulations, as compared to the control group (*p* < 0.05). Several *Bacillus* species have been reported to reduce the severity of disease on a range of hosts (Kim et al., 2012; Lee et al., 2013). Similarly, Shin et al. (2021) demonstrated that the application of the antagonistic bacteria, *B. velezensis* BS1 at a concentration of 10^5^ × CFU/mL significantly (*p* < 0.05) reduced the anthracnose disease caused by *Colletotrichum scovillei* on hot pepper fruits when compared to the untreated control group. Furthermore, Kwon et al. (2022) achieved red pepper anthracnose control using *Bacillus tequilensis* GYUN-300 with only 14% disease incidence under field conditions. In contrast, Kim et al. (2023) recently demonstrated disease control of anthracnose caused by *C. acutatum* in red peppers with a significant (*p* < 0.05) reduction of disease rate (0.4%) by pre-immersion with *Brevibacillus halotolerans* B-4359 when compared to foliar spray and soil drench methods under field conditions. Nevertheless, several factors, such as environmental conditions, soil microorganisms, and pathogenesis, impact the disease incidence in field conditions (Wei et al., 2015).

Our study further reports the complete genome sequence of the strain GYUN-2311, comprising 4,096,969 bp on the chromosome. Using whole-genome analysis, the genome of the GYUN-2311 strain was compared to those of analogous species within the same genus. *Bacillus* spp possess non-ribosomal peptide (NRP) gene clusters that produce cyclic peptides with antimicrobial properties, including bacillibactin, brevicidine, and brevicidine B (Zhao et al., 2019; Zhao and Kuipers, 2021). In particular, variations in biocontrol targets and efficacy may result from differences between GYUN-2311 and other *Bacillus* strains due to biocontrol-related genes and gene clusters implicated in antibiotic resistance. This result suggests that the strain GYUN-2311 employed its antifungal activity by the production of these antibiotics. The genome of strain GYUN-2311 was analyzed with antiSMASH to detect for the presence of secondary metabolites; eight clusters of genes encoding NRP biosynthesis were identified as secondary metabolite genes. Except for surfactin, all other peptides, including subtilomycin, bacillaene, fengycin, bacillibactin, subtilosin, and bacilysin were identified as 100% in contig 2; these peptides play a role in the suppression of pathogens by inducing systemic resistance (Kamali et al., 2022). In a recent report by Thiruvengadam et al. (2022), genomic analysis of the strain *B. subtilis* Bbv57 isolated from betelvine rhizosphere sediments revealed 9 putative biosynthetic secondary metabolite gene clusters.

*Bacillus subtilis* has been reported to produce several antibiotics, such as subtilosin A, surfactin, and fengycin, which can be used to control plant diseases caused by bacterial and fungal pathogens (Falardeau et al., 2013; Mora et al., 2015). The GYUN-2311 genome comprises eleven gene clusters that are involved in the biosynthesis of secondary metabolites, including subtilomycin, surfactin, bacillaene, fengycin, bacillibactin, pulcherriminic acid, subtilosin A, and bacilysin. In addition, we found that genes involved in the production of secondary metabolites are expressed in GYUN-2311 and that their expression is enhanced in the presence of pathogenic fungi in a dual culture system, because they are more sensitive toward the pathogen. For instance, Liang et al. (2023) observed that the antibiotic biosynthesis and metabolism were enriched among down-regulated genes in dual culture of *Bacillus velezensis* E68 and *F. graminearum* DAOMC 180378. The expression of surfactin gene increased 2.83-fold when compared to the monoculture of GYUN-2311. Surfactins are widely known for their antiviral and antibacterial properties (Ongena and Jacques, 2008). Particularly, surfactins have the ability to combat major plant diseases (Lee et al., 2007; Romano et al., 2013). When GYUN-2311 is cultured, various secondary metabolites are secreted into the culture, exhibiting antimicrobial activity in the CF, especially when cultured in LB medium, with an antimicrobial activity of 81.4% against *B. dothidea*.

In conclusion, the strain GYUN-2311 isolated from apple rhizosphere soil demonstrated significant antagonistic activity against multiple fungal pathogens, including *C. siamense* and *C. acutatum*, which cause anthracnose in apples and hot peppers, respectively. GYUN-2311 cell suspensions inhibited conidia germination and appressorium formation to suppress anthracnose *in vitro*. This strain inhibited apple and hot pepper anthracnose *in vivo* and produced hydrolytic enzymes *in vitro*. The strain has been found to promote the plant growth of hot peppers in greenhouse conditions. It was demonstrated that hot pepper anthracnose could be suppressed in the field in Andong, Gyeongbuk Province, South Korea, using GYUN-2311 suspension treatment. According to whole-genome sequencing, the core genome of GYUN-2311 exhibited a similarity of 100% to that of *B. subtilis* SRCM104005. Based on the whole-genome sequencing analysis, the strain GYUN-2311 was determined as *B. subtilis*. During the whole-genome sequencing of strain GYUN-2311, antimicrobial secondary metabolites including subtilomycin, bacillaene, fengycin, bacillibactin, subtilosin, and bacilysin were identified. Our findings imply that GYUN-2311 could be a potential BCA for ecologically friendly phytopathogen control. Future investigations will utilize proteomic and transcriptome techniques to examine the signaling pathways that are associated with the deleterious impacts of secondary metabolites.

## Data availability statement

The datasets presented in this study can be found in online repositories. The names of the repository/repositories and accession number(s) can be found in the article/Supplementary material.

## Author contributions

YH: Funding acquisition, Investigation, Methodology, Writing—original draft. YL: Investigation, Methodology, Data curation, Writing—review and editing. KB: Data curation, Writing—original draft, Writing—review and editing. YJ: Writing—review and editing, Conceptualization, Supervision, Validation.

## Footnotes

1
https://www.pacb.com/

2
https://www.EZbiocloud.net

3
http://eggnogdb.embl.de

4
https://dl.secondarymetabolites.org/releases/7.0.0/
