# Supplementary material for: Characterization and evaluation of Bacillus subtilis GYUN-2311 as a biocontrol agent against Colletotrichum spp. on apple and hot pepper in Korea
Source: Front Microbiol. 2024 Jan 8;14:1322641. doi: 10.3389/fmicb.2023.1322641 (PMC10800898; doi:10.3389/fmicb.2023.1322641)
Supplement: Supplementary file 1 [file Data_Sheet_1.pdf]

Supplementary information

Characterization and evaluation of *Bacillus subtilis* GYUN-2311 as a biocontrol agent against *Colletotrichum* sp. on apple and hot pepper

Yunjeong Heo[1], Younmi Lee[1], Kotnala Balaraju[2], Yongho Jeon[1]*

[1]Department of Plant Medicals, Andong National University, Andong 36729, Republic of Korea

[2]Agricultural Science & Technology Research Institute, Andong National University, Andong 36729, Republic of Korea

*Corresponding author:

Prof. Yongho Jeon

E-mail: yongbac@andong.ac.kr

Tel: +82-54-820-5507

Fax: +82-54-820-6320

Title: Characterization of *Bacillus subtilis* GYUN-2311

Supplementary Tables

Supplementary Table 1. The fungal pathogen used in the study

	Pathogen Species	Strain	Abbreviation	Disease	Host	Origin or Collection
1	*Colletotrichum acutatum*	KACC42403	*Ca*	Anthracnose	Hot pepper	KACC
2	*Colletotrichum coccodes*	ACC48737	*Cc*	Anthracnose	Hot pepper	KACC
3	*Colletotrichum siamense*	YJHR001	*Cs*	Bitter rot (Anthracnose)	Apple	Lab. Strain
4	*Colletotrichum fructicola*	YJFJ007	*Cfr*	Bitter rot (Anthracnose)	Apple	Lab. Strain
5	*Colletotrichum gloeosporioides*	YYFJ005	*Cg*	Bitter rot (Anthracnose)	Apple	Lab. Strain
6	*Colletotrichum aenigma*	YDDF2	*Cae*	Bitter rot (Anthracnose)	Apple	Lab. Strain
7	*Colletotrichum fioriniae*	ANYA4	*Cfi*	Bitter rot (Anthracnose)	Apple	Lab. Strain
8	*Colletotrichum nymphaea*	BHBF13	*Cn*	Bitter rot (Anthracnose)	Apple	Lab. Strain
9	*Diplodia seriata*	DS1	*Ds*	Bitter rot (Anthracnose)	Apple	Lab. Strain
10	*Botryosphaeria dothidea*	BWFJ22	*Bd*	White rot/Dieback	Apple	Lab. Strain
11	*Fusarium solani*	GSF3	*Fs*	Root rot	Ginseng	Lab. Strain
12	*Fusarium oxysporum* f. sp. *lycopersici*	KACC40043	*Fo*	Wilt	Tomato	KACC

Supplementary Table 2. The detailed treatment schedule

Treatment/Date	7/7	7/20	7/31	8/14	8/22
Control	-	-	-	-	-
Systemic chemical	Acibenzolar-s-methyl+mancozeb	Propineb	Pyraclostrobin	Chlorothaloniil + Difenoconazole	Dithianon + Pyraclostrobin
Pyraclostrobin (negative control)	Pyraclostrobin	Pyraclostrobin	Pyraclostrobin	Pyraclostrobin	Pyraclostrobin
TK®	TK®	TK®	TK®	TK®	TK®
GYUN-2311	GYUN-2311	GYUN-2311	GYUN-2311	GYUN-2311	GYUN-2311
GYUN-2311 + chemical (CRS)	GYUN-2311	Propineb	GYUN-2311	Chlorothaloniil + Difenoconazole	GYUN-2311
GYUN-2311 + chemical (MIS)	GYUN-2311 + Acibenzolar-s-methyl+mancozeb	GYUN-2311 + Propineb	GYUN-2311 + Pyraclostrobin	GYUN-2311 + Chlorothaloniil + Difenoconazole	GYUN-2311 + Dithianon + Pyraclostrobin

Supplementary Table 3. qRT-PCR primers (F: forward primer, R: reverse primer) and product length

Gene	Sequence (5'-3')	Product size (bp)
bacillaene	F: GACGTGGTCCAACAGGTCAT	218
	R: GTTTTCACCGACCCAATGCC	
bacillibactin	F: ACCTGACAAACGGACAGCAA	191
	R: TTGACGAACCGACAGTAGCC	
fengycin	F: ATAACCTCTGGCAACACCCG	174
	R: GACGCGGCATTTTACGTGTT	
subtilomycin	F: TCGATTACCTGGGTTGGTGC	207
	R: ATACAGAACGATGTCGGCCC	
subtilosin A	F: AAAAGGTTCGGGATCTCGGG	189
	R: CCGCGCGTTTTCTCTCATAG	
surfactin	F: AGTGCTTGCTGCAACGAATG	189
	R: TGTTTTCGGTCCGATCCCTG	
16S rRNA (Accession no. AB042061; Gao et al., 2011)	F: TCGCGGTTTCGCTGCCCTTT	177
	R: AAGTCCCGCAACGAGCGCAA	

Supplementary Figures

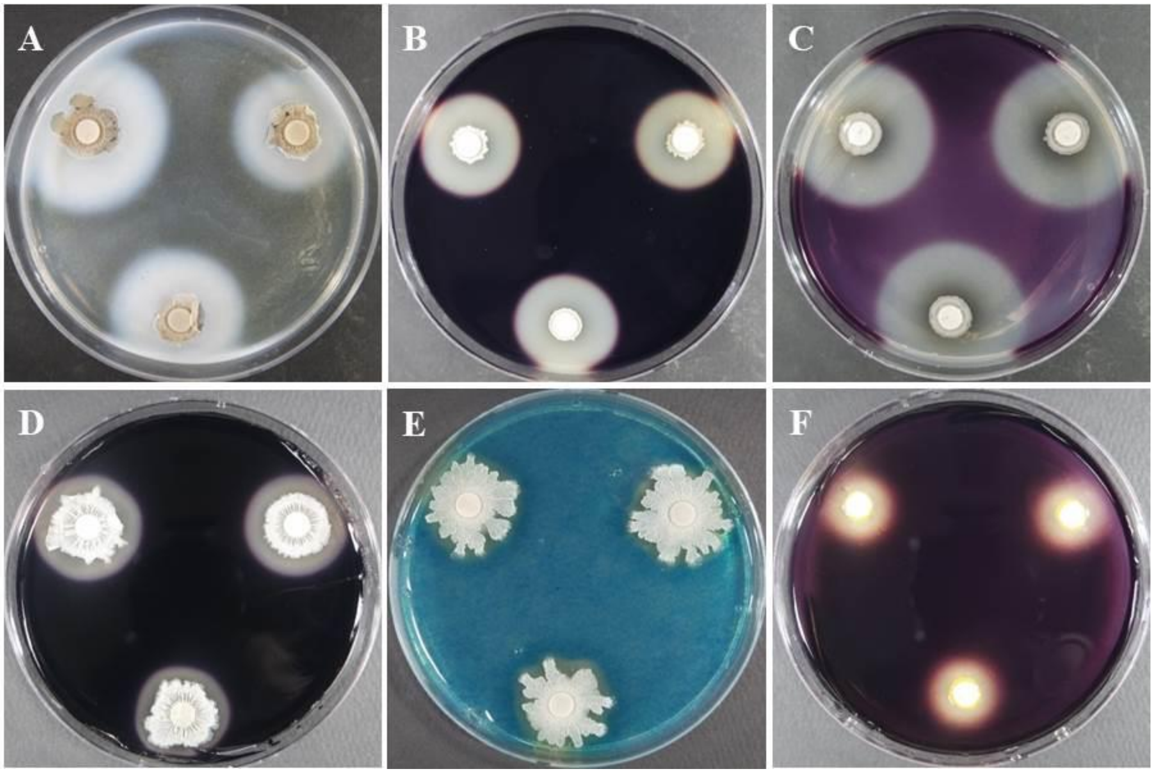

Supplementary Figure 1. Characterization of antagonistic substances produced by GYUN-2311 strain. Detection of protease (a), cellulase (b), chitinase (c), and amylase (d) by *B. subtilis* GYUN-2311. Siderophore production (e) and inorganic phosphate solubilization (f) assay *in vitro* by GYUN-2311.

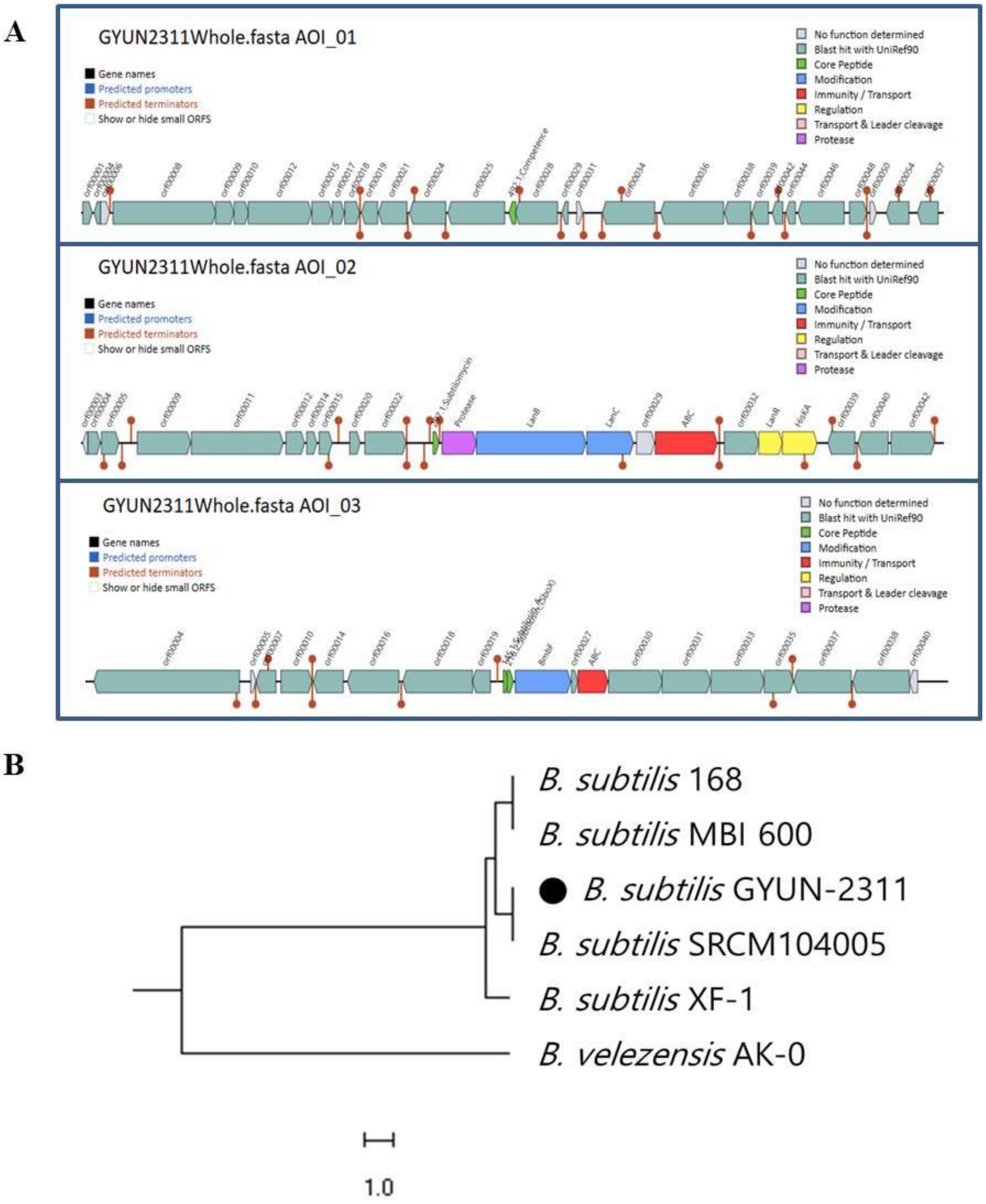

Supplementary Figure 2. GYUN-2311 genome analysis by BAGEL 4.0. (A) According to the analysis, the GYUN-2311 genome contains three gene clusters with the potential to synthesize competence (AOI_01; top), subtylomycin (AOI_01; middle), and subtilosin A (AOI_03; bottom). (B) ANI-derived UPGMA dendrogram (Newick format).
